# Survival after postoperative morbidity: a longitudinal observational cohort study^†^

**DOI:** 10.1093/bja/aeu224

**Published:** 2014-07-10

**Authors:** S. R. Moonesinghe, S. Harris, M. G. Mythen, K. M. Rowan, F. S. Haddad, M. Emberton, M. P. W. Grocott

**Affiliations:** 1 UCL/UCLH Surgical Outcomes Research Centre, Department of Anaesthetics, University College Hospital, London NW1 2BU, UK; 2 UCL Centre for Anaesthesia, University College Hospital, London NW1 2BU, UK; 3 National Institute for Academic Anaesthesia's Health Services Research Centre, Royal College of Anaesthetists, 35 Red Lion Square, London WC1R 4SG, UK; 4 London School of Hygiene and Tropical Medicine, Keppel Street, London WC1E 7HT, UK; 5 Intensive Care National Audit & Research Centre, Napier House, 24 High Holborn, London WC1 V 6AZ, UK; 6 Institute of Sports, Exercise and Health, University College London, Gower Street, London WC1E 6BT, UK; 7 Division of Surgery and Interventional Science, University College London, Gower Street, London WC1E 6BT, UK; 8 Integrative Physiology and Critical Illness Group, University of Southampton, Southampton, UK; 9 Anaesthesia and Critical Care Research Unit, University Hospital Southampton NHS Foundation Trust, Southampton, UK

**Keywords:** complications, complications, morbidity, complications, neurological, surgery, non-cardiac

## Abstract

**Background:**

Previous studies have suggested that there may be long-term harm associated with postoperative complications. Uncertainty exists however, because of the need for risk adjustment and inconsistent definitions of postoperative morbidity.

**Methods:**

We did a longitudinal observational cohort study of patients undergoing major surgery. Case-mix adjustment was applied and morbidity was recorded using a validated outcome measure. Cox proportional hazards modelling using time-dependent covariates was used to measure the independent relationship between prolonged postoperative morbidity and longer term survival.

**Results:**

Data were analysed for 1362 patients. The median length of stay was 9 days and the median follow-up time was 6.5 yr. Independent of perioperative risk, postoperative neurological morbidity (prevalence 2.9%) was associated with a relative hazard for long-term mortality of 2.00 [*P*=0.001; 95% confidence interval (CI) 1.32–3.04]. Prolonged postoperative morbidity (prevalence 15.6%) conferred a relative hazard for death in the first 12 months after surgery of 3.51 (*P*<0.001; 95% CI 2.28–5.42) and for the next 2 yr of 2.44 (*P*<0.001; 95% CI 1.62–3.65), returning to baseline thereafter.

**Conclusions:**

Prolonged morbidity after surgery is associated with a risk of premature death for a longer duration than perhaps is commonly thought; however, this risk falls with time. We suggest that prolonged postoperative morbidity measured in this way may be a valid indicator of the quality of surgical healthcare. Our findings reinforce the importance of research and quality improvement initiatives aimed at reducing the duration and severity of postoperative complications.

**Editor's key points**

- Postoperative morbid events can have long-term implications for some patients.
- Which specific complications affect patient disability-free survival and wellbeing remain largely unknown.
- An ageing surgical population and growing demands for cost-effective healthcare have placed greater scrutiny on patient-centred measures of outcome.
- Clinical epidemiology of the entire perioperative pathway, including late outcomes, is an important component of this process.

Surgical morbidity and mortality is a significant public health issue. Worldwide, it has been estimated that more than 230 million major surgical procedures take place each year,^1^ and we know that there is a substantial international variation in postoperative morbidity^2^ and mortality.^3^ Estimates of postoperative morbidity vary between 7%^4^ and 50%,^5^ depending on the type of surgery, patient risk factors, and on how complications are defined.^6^^7^

Moreover, the health implications of the surgical insult may be sustained. The ‘Whitehall study’, which followed more than 6000 British civil servants for a median of 10 yr, found that absence from work of >7 days after surgery was one of the most significant risk factors for reduced long-term survival, second only to work-absence because of circulatory disease.^8^ Long-term consequences of postoperative morbidity were reported in a study of more than 100 000 patients who underwent eight different types of major surgery, conducted by the American College of Surgeons' National Surgical Quality Improvement Programme. The investigators observed an association between the occurrence of complications within 30 days of operation and increased long-term mortality (average follow-up time 8 yr).^9^ This study used more robust case-mix adjustment than the Whitehall study to account for perioperative risk factors. However, close inspection of survival curves shows that the risk of long-term harm may vary with time after surgery; this possible variation was not explored in the report.

To better understand the relationship between postoperative morbidity and risk of premature death, we linked two prospective surgical cohort studies with long-term survival in which validated outcome measures were used, risk adjustment applied, and in which time was used as an interaction variable, in order to test the hypothesis that short-term postoperative morbidity is independently associated with reduced longer term survival.

## Methods

The primary objective of this prospective observational cohort study was to measure the independent relationships between perioperative risk, inpatient postoperative morbidity, and long-term survival. Patients undergoing elective, non-cardiac, non-neurological surgery that was classified as ‘major’ were recruited between the years 2001 and 2005 at the Middlesex Hospital, London, UK. The patients comprised two separate cohorts. In Cohort 1, 438 patients were recruited into a prospective observational study conducted by the UCL/UCLH Surgical Outcomes Research Centre (SOuRCe), which validated the Post Operative Morbidity Survey (POMS) for the first time.^6^ Patients within Cohort 2 were participants in a service evaluation of the departments of surgery and anaesthesia at the same institution between March 2004 and April 2005. Both studies underwent ethics review and received approval from the Joint UCLH/UCL Committee on the Ethics of Human Research. Inclusion and exclusion criteria for both studies are listed in Supplementary Appendix S1.

All patients enrolled into the original studies were followed up using the UK Medical Research Information Service (MRIS), which provided the date of exit from the National Health Service for patients who left the UK, and for deceased patients, date of death. The Ethics and Confidentiality Committee of the National Information Governance Board granted approval for disclosure of follow-up data in June 2009 (Study Number MR1152).

### Dataset

The following data were collected on all patients during the original study periods: age, surgical procedure, surgical specialty, and measures of perioperative risk: the American Society of Anesthesiologists' Physical Status Score (ASA-PS)^10^ and the variables of the Portsmouth Physiology and Operative Severity Score for the enUmeration of Morbidity and mortality (P-POSSUM).^11^ P-POSSUM consists of 12 physiological variables (including cardiac and respiratory history, and a number of haematological and biochemical parameters) and six operative variables (including urgency and severity of procedure, malignancy status, and blood loss). Postoperative morbidity was measured using the POMS, a validated measure of postoperative harm,^6,12^ administered on Days 3, 5, 8, and 15 after surgery. The length of postoperative stay was also recorded. Two study nurses, who had received training in how to identify and interpret the preoperative risk and postoperative outcome variables within the dataset, collected all data prospectively. Data linkage between the SOuRCe dataset and the MRIS at the NHS Information Centre provided date of death for non-surviving cohort members. The study closed on March 1, 2012.

### Statistical analysis

#### Data presentation and management

Continuous data are presented as mean and standard deviation (sd), or median and inter-quartile range (IQR) when not normally distributed. Categorical data are expressed as percentage and counts. Missing data were not imputed and models were based on complete cases.

Mortality is summarized at hospital discharge, 30 days, 1 and 5 yr after operation date. Morbidity is described as a dichotomous outcome using the following definitions: any inpatient morbidity (POMS defined morbidity at any stage), POMS domain defined (separate dichotomous outcomes for each of the nine domains), POMS day defined (separate dichotomous outcomes for presence or absence of POMS defined morbidity on each of Days 3, 5, 8, and 15 after surgery).

Dichotomous variables were assessed using the *χ*^2^ testing with Bonferroni's correction for multiple comparisons. The discrimination of the P-POSSUM model as a perioperative risk adjuster was tested by measuring the area under the receiver-operator-characteristic curve for inpatient mortality.

### Survival analysis

Time zero was the date of operation; right censoring occurred on March 1, 2012. Patients who died within 15 days of surgery were excluded from survival analyses, as Day 15 POMS status was included as an explanatory variable.

Kaplan–Meier plots were used to measure the relationship between categorical variables and long-term survival. Variables associated with survival using the log-rank test with *P*<0.05 were included in the initial multi-variable model. P-POSSUM-predicted mortality was entered as a continuous variable. Although age is a variable within the P-POSSUM score, it was also separately included as a continuous variable in the analysis, as it was hypothesized that the effect size of advanced age would be somewhat greater in a long-term survival analysis than in a short-term mortality model. Similarly, although cancer status (based on surgical data) is also included with the P-POSSUM, cancer status was included as a separate binary indicator variable in the long-term survival analysis. The type of postoperative morbidity was entered as separate indicator variables for each of the POMS domains that were significantly associated with long-term survival on univariate analysis. Duration of morbidity was defined by the final day on which POMS-defined morbidity was recorded [final morbidity day (FMD)] and was included as multiple dichotomous variables. The POMS has not been validated as a ‘score’ (i.e. there is insufficient internal consistency between domains to be able to use the POMS as a scale where the number of positive domains is representative of the severity of postoperative complications).^6^ Therefore, we did not measure the relationship between the number of POMS positive domains and long-term outcome. We also tested for interactions between gastrointestinal (GI) morbidity and general surgery, and cardiac morbidity and vascular surgery. We tested for, and excluded, collinear variables before model construction. After constructing the initial model, the proportional hazards (PH) assumption was tested using Schoenfeld's partial residuals and the result confirmed by visual examination of a log-minus-log survival plot.

Model development was based on stepwise significance testing; initially variables were dropped based on *P* values >0.10 then *P*>0.05. Model fit was assessed with the likelihood ratio (LR) testing. All statistical analyses were conducted using Stata InterCooled (Release 12.1) software (StataCorp LP, College Station, TX, USA).

## Results

### General description

Data were available for 1362 patients, 438 in Cohort 1 and 924 in Cohort 2. The cohorts were similar in age, gender, ASA-PS score, and the distribution of surgical specialities; however, the first cohort was higher risk [P-POSSUM-predicted mortality mean (sd) 2.52 (5.36) *vs* 1.65 (2.76); *P*<0.001]. The median length of hospital stay was 9 days (IQR: 6–14). The general characteristics of the study population are shown in Table 1. The relationship between FMD and postoperative length of stay is shown in Table 2. The incidence of postoperative morbidity in different types of surgery is shown in Table 3.

**Table 1:** Baseline patient characteristics

Variable	
Number	1362
Mean age (sd)	63.5 (15.3)
Female gender (%)	773 (56.8)
Ethnicity: *n* (%)	
Black or Black British	69 (5.1)
White or White British	1198 (88.0)
Asian or Asian British	44 (3.2)
Other	49 (3.6)
Missing	2 (0.2)
P-POSSUM predicted mortality	
Mean (sd)	1.9 (3.8)
Median (IQR)	1.0 (0.6–1.8)
Surgical Specialty: *n* (%)	
Orthopaedic	855 (62.8)
General	296 (21.7)
Urology	147 (10.8)
Vascular	64 (4.7)
ASA-PS class: *n* (%)	
I	223 (16.4)
II	808 (59.3)
III	299 (22.0)
IV	13 (1.0)
Missing	19 (1.4)

**Table 2:** Relationship between FMD and postoperative length of stay (days)

Postoperative length of stay (days)	Total cohort (*n*=1362)	No morbidity (*n*=368)	FMD 3 (*n*=266)	FMD 5 (*n*=278)	FMD 8 (*n*=238)	FMD 15 (*n*=212)
Median (IQR)	9 (6–14)	6 (4–7)	7 (6–9)	8 (7–11)	13 (11–15)	25 (19.5–36)

**Table 3:** Incidence of postoperative morbidity according to surgical specialty (%)

	Total (*n*=1362)	Orthopaedic (*n*=855)	General (*n*=296)	Urology (*n*=147)	Vascular (*n*=64)
Any morbidity	73.0	62.9	91.0	91.1	82.8
Any pulmonary	33.1	22.6	52.4	50.3	45.3
Any infection	40.3	32.4	47.3	64.6	57.8
Any renal	43.6	31.1	59.5	76.2	62.5
Any GI	41.0	23.9	83.8	55.1	39.1
Any cardiac	6.8	5.6	7.4	4.8	23.4
Any neurological	2.9	2.5	2.0	4.8	9.4
Any wound	11.0	11.1	11.5	9.5	10.9
Any haematology	8.1	7.8	6.8	10.9	10.9
Any pain	33.4	20.1	60.8	53.7	37.5
POMS+ Day 3	67.8	55.6	90.2	90.5	75.0
POMS+ Day 5	49.9	35.8	77.4	70.1	64.1
POMS+ Day 8	31.5	20.9	53.0	44.9	42.2
POMS+ Day 15	15.6	8.2	29.4	26.5	25.0

Long-term follow-up data were analysed for 1342 patients; the reasons for 20 exclusions were: missing follow-up data (*n*=13) and death within 15 days of surgery (*n*=7). The majority of patients (79%) was undergoing non-cancer surgery and had no evidence of malignancy at the time of surgery. There were 383 deaths in the final group (28.1%); maximum duration of follow-up was 3895 days (10.7 yr); median follow-up was 2375 days (6.5 yr; IQR 2696–2899 days). Mortality at hospital discharge, 30 days, 1, and 5 yr is summarized in Table 4. The area under the receiver-operator-characteristic curve for P-POSSUM score for predicting inpatient mortality was 0.85 [standard error (se) 0.03; 95% confidence interval (CI) 0.78–0.91].

**Table 4:** Comparison of postoperative mortality (%) according to surgical specialty. *With Bonferroni's correction for four analyses

	Inpatient (*n*=1362)	30 day (*n*=1362)	1 yr (*n*=1347)	5 yr (*n*=1339)
All patients	1.5	1.1	6.8	20.7
Orthopaedics	0.5	0.6	3.0	13.4
Urology	1.4	0	6.2	18.3
General	3.4	2.4	16.4	40.4
Vascular	7.8	4.7	15.9	32.8
*P*-value*	<0.001	<0.01	<0.001	<0.001

### Univariate analysis

The occurrence of postoperative morbidity, of any aetiology defined by POMS, was associated with reduced long-term survival (*P*<0.001). Log-rank comparisons of survival between patients who did and did not develop pulmonary, renal, infectious, GI, neurological, cardiac, and pain morbidity all showed differences at *P*<0.001. There was also a difference in survival between patients who were positive and negative for haematological morbidity (*P*=0.002). There was no difference in long-term outcome based on the development of wound morbidity (*P*=0.42).

### Multi-variable analysis

**Fig 1:**
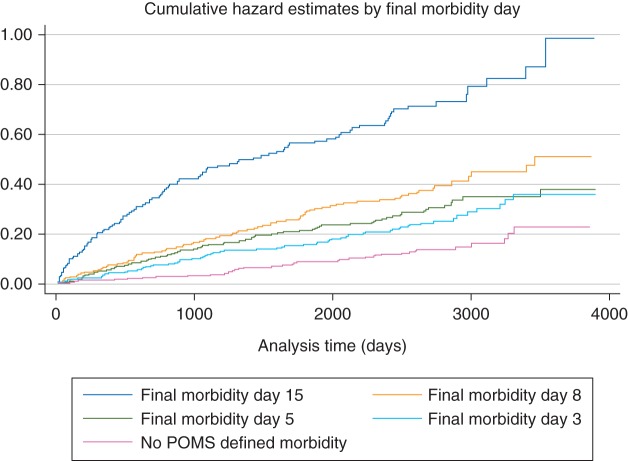
Cumulative hazard plot for mortality after postoperative morbidity according to FMD.

When a model was initially constructed and the PH assumption tested, both tests showed that the PH assumption was violated. Plotting baseline cumulative hazard for patients with different durations of postoperative morbidity demonstrated a ‘step’ in the cumulative hazard at ∼3 yr after operation in patients whose FMD was Day 15. No such change in trajectory was seen for the patients who either did not have any postoperative morbidity recorded, or whose FMD was 3, 5, or 8 (see Fig. 1). Therefore, time was included as an interaction with FMD 15, in order to avoid the need to comply with the PH assumption.^13,14^ The follow-up duration was split into three periods: 0–365, 366–1095, >1095 days (3 yr). The unrestricted model included the interactions of each of these time categories with FMD 15.

The full model therefore consisted of 27 variables; stepwise analyses led to a final model of 11 variables based on significance testing; this model is listed in Table 5. The LR *χ*^2^ test for the final model was 292.7, giving a *P*-value of <0.0001, thus demonstrating that the unrestricted model was not different from the final model.

**Table 5:** Final Cox PH model for long-term mortality

Variable	Hazard ratio	se	*P*-value	95% CIs
P-POSSUM percentage risk	1.05	0.01	<0.001	1.03–1.07
Cancer	2.71	0.39	<0.001	2.05–3.58
Age (yr)	1.03	0.00	<0.001	1.02–1.04
General surgery	1.46	0.21	<0.01	1.10–1.93
Vascular surgery	2.34	0.48	<0.001	1.56–3.50
Any neurological morbidity	2.00	0.43	<0.01	1.32–3.04
FMD 15+ postoperative year 1	3.51	0.78	<0.001	2.28–5.42
FMD 15+ postoperative years 2–3	2.44	0.50	<0.001	1.62–3.65

The perioperative risk factors of age, cancer history, P-POSSUM predicted risk, and the procedure categories of general or vascular surgery were all independently associated with reduced long-term survival. In addition, the occurrence of neurological morbidity (prevalence 2.94%) after surgery was associated with a relative hazard for long-term mortality of 2.00 (*P*=0.001; 95% CI 1.32–3.04). Morbidity which persisted until at least Day 15, and thus associated with a greater than average length of stay within this cohort, was associated with a relative hazard of 3.51 (95% CI 2.28–5.42) for the first year after surgery, 2.44 for the next 2 yr (95% CI 1.62–3.65), and returning to baseline thereafter.

## Discussion

This study shows that in major elective, non-cardiac, non-neurological surgery, prolonged postoperative morbidity (as defined by the POMS) is independently associated with an increased risk of death for up to 3 yr after surgery. This reiterates the association shown by Khuri and colleagues^9^ and others^15^ but using different approaches to measurement of both risk and morbidity (complications); this therefore increases the likelihood that this relationship may be causal.^16^ We believe that this is the first study to demonstrate that this increased risk varies with time, returning to baseline after 3 yr. The findings of this study should invigorate current efforts to make surgery safer. Furthermore, we suggest that prolonged postoperative morbidity is a valid measure of the quality of surgical healthcare, which, when adjusted for perioperative risk, would be a useful outcome measure for clinical effectiveness studies and for comparative audit.

### Methodological limitations

Before putting these results into context, it is worth addressing some methodological issues. Our study was conducted in a single centre and recruited solely elective patients, and as a result there are limits to the generalizability of our findings. No adjustment was made for the effect that social deprivation might have had on long-term outcome: life expectancy is strongly related to index of deprivation^17^ and therefore this may have influenced postoperative survival. The risk adjustment was confined by the limitations of the original SOuRCe dataset, and there are risk factors for both perioperative and long-term outcome that were not included in the model (e.g. diabetes mellitus). It is therefore possible that the case-mix adjustment was not as complete as possible and that the presence of latent confounding may have led to an overestimation of the association between postoperative morbidity and long-term outcome. However, the P-POSSUM model has been widely validated as a perioperative risk adjuster,^18^ and in this population, we found it to be highly accurate for the prediction of short-term mortality, therefore justifying its use. Finally, there are also limitations of the POMS as an outcome measure. It is highly sensitive: it was developed to detect morbidity of a type that would prolong hospital stay; therefore, POMS definitions encompass some relatively minor and process-related morbidity, such as the presence of a urinary catheter. This may explain the seemingly high morbidity figures, particularly on postoperative day 3, when it is perhaps more fair to say that POMS reflects the ‘absence of full recovery’ defined by the ongoing delivery of care, rather than ‘true’ morbidity.^6^ However, the morbidity rates for the latter days are broadly similar to previous reports using the POMS tool in similar patient cohorts.^12^

### Clinical implications

Our data support the notion that the occurrence of postoperative morbidity is a major public health issue, which (in terms of adjusted risk) is comparable in scale with the long-term consequences of obesity^19^ and diabetes mellitus.^20^ However, unlike the harm associated with these diseases, the long-term risk, we have described may be more amenable to prevention, both through progress in our understanding of the pathophysiology of adverse surgical outcomes and via improvement in the quality of surgical healthcare. For example, we have demonstrated an association between postoperative neurological morbidity and the risk of reduced long-term survival; previous studies have also found this link.^21^ Postoperative cognitive dysfunction and delirium is a fairly common event, particularly in the elderly.^22,21^ Studies in animal models have pointed to a plausible immunological mechanism for the development of postoperative cognitive decline, which is triggered by the physiological response to injury; this immune response could serve as a target for therapy.^23,24^

Additionally, it is an important observation that the majority of patients in this cohort were undergoing joint replacement surgery, which is aimed at improving quality of life, as opposed to, for example, cancer surgery, aimed at prolongation of life. The implications of prolonged postoperative morbidity and the increased risk of premature death are particularly important in this sub-group of patients: understanding and quantifying this potential risk should inform the consent process and potentially influence the views of both the patient and clinical team when making decisions on whether to undertake surgery in higher risk patients.

### Prolonged postoperative morbidity as a quality metric

Short-term postoperative morbidity and mortality continues to vary across providers and healthcare systems.^3^^25,26^ While it is true that some complications may be unavoidable, particularly in patients with multiple co-morbidities, it is also true that prolonged morbidity, major complications, and ultimately death may be mitigated against by improved structures and processes in healthcare: this ‘failure-to-rescue’ concept has widely been studied in the USA.^26,27^ Wider implementation of strategies such as goal-directed fluid therapy,^28,29^ enhanced recovery (or ‘fast-track surgery’) programmes,^30^ and the expansion of critical care facilities so that a greater number of high-risk patients can be managed in high-acuity ward areas^3^ may contribute to a reduction in the disease burden that arises from the development of postoperative complications.

Currently, mortality and the length of stay are two of the most commonly measured postoperative outcomes; however, both have limitations. Although there are some high-risk procedures which provide exceptions,^31^ overall surgical mortality is low in high-resource nations. Thus, in order to be able to make meaningful comparisons between institutions and teams, an outcome measure with a higher prevalence (such as complication rates) is required.^32,33^ Furthermore, while the length of stay is an attractive quality metric, as it is easy to measure, it is also prone to biases that are not related to true patient outcome or the quality of care, such as socioeconomic status.^34^

### Longer term impact of prolonged postoperative morbidity: potential hypotheses

Our study challenges previous temporal associations—and therefore potential mechanistic explanations—for the relationship between non-specific postoperative morbidity and longer term risk of death. We believe that by using time-dependent covariates, our work is the first to demonstrate that while the impact of prolonged postoperative morbidity on survival is of greater duration than may be intuitive, that this risk falls progressively until reaching baseline after 3 yr. This long tail suggests that the underlying mechanism conferring the risk must persist for a considerable period of time, and possibly for long after the resolution of clinically apparent morbidity. Currently, the most plausible explanation—and one that has garnered considerable support—is that the agent conferring the risk is inflammation.^35^ If inflammation does prove to be the underlying process leading to long-term risk, then this too should be amenable to mitigation by either changing practices or testing anti-inflammatory therapies.^36^

As with all epidemiological data, our results should be regarded as hypothesis generating rather than confirmatory. From these data, we are unable to prove that some or all of the prolonged postoperative morbidity might have been related to the quality of inpatient care and therefore might have been avoidable. Unmeasured confounding from patient comorbidities may account for some of the total effect, which leads to prolonged morbidity; however, it is unlikely that patient-related risk alone is the sole determinant of outcome: our study recapitulates the findings of others from large observational cohorts and ‘real-world’ audit data and using a variety of risk and outcome measures. Furthermore, we cannot assume that by preventing prolonged postoperative morbidity that long-term survival would improve; similar confounders may influence the longer term disease trajectory. However, our study provides important data that may be used to support future work evaluating the underlying mechanisms of postoperative morbidity and strategies which might be targeted at mitigating risk, either before surgery, or after discharge from hospital.

In conclusion, we have demonstrated that postoperative neurological morbidity is associated with reduced survival in the longer term, and that prolonged postoperative morbidity of any aetiology is associated with increased mortality risk for up to 3 yr after the surgical insult. We suggest that prolonged postoperative morbidity is a valid and important quality metric for the evaluation of perioperative care. Our findings support the need for future work directed at implementing structure and process-related changes in healthcare delivery in order to reduce postoperative morbidity.

## Authors’ contributions

The study idea was conceived by S.R.M. and M.P.W.G. The original study data were prospectively collected by the Surgical Outcomes Research Centre (SOuRCe) research nurses, under the supervision of M.G.M., M.P.W.G., F.S.H., and M.E. S.R.M. was responsible for ethics, data linkage, cleaning, and analysis, with input into the analysis from S.H. The manuscript was drafted by S.R.M. and subsequently revised after critical review by all authors.

## Supplementary material

Supplementary material is available at *British Journal of Anaesthesia* online.

## Declaration of interest

None declared.

## Funding

S.R.M., M.G.M., F.S.H., and M.E. work within the University College Hospitals Foundation Trust/University College London National Institute for Health Research (NIHR) Comprehensive Biomedical Research Centre, which received a portion of funding from the UK Department of Health Biomedical Research Centres funding scheme. S.H. was a Wellcome Foundation research fellow until 2013 and is currently an NIHR Academic Clinical lecturer. M.P.W.G. works within the University Hospital Southampton NHS Foundation Trust/University of Southampton NIHR Respiratory Biomedical Research Unit, which received a portion of funding from the UK Department of Health Biomedical Research Units funding scheme.

## Supplementary Material

Supplementary Data
