# Supplementary material for: Survival after postoperative morbidity: a longitudinal observational cohort study
Source: Br J Anaesth. 2014 Jul 10;113(6):977–84. doi: 10.1093/bja/aeu224 (PMC4235571; doi:10.1093/bja/aeu224)
Supplement: Supplementary Data [file supp_113_6_977__index.html]

Survival after postoperative morbidity: a longitudinal observational cohort study — Supplementary Data 

# Survival after postoperative morbidity: a longitudinal observational cohort study

## Supplementary Data

Supplementary Data

**Files in this Data Supplement:**

- Supplementary Appendix 1 - docx file
- Supplementary Appendix 2 - docx file
