# Supplementary material for: Survival after postoperative morbidity: a longitudinal observational cohort study
Source: Br J Anaesth. 2014 Jul 10;113(6):977–84. doi: 10.1093/bja/aeu224 (PMC4235571; doi:10.1093/bja/aeu224)
Supplement: Supplementary Data [file supp_aeu224_aeu224supp_app1.docx]

## Appendix One: Inclusion criteria

### Cohort 1

Major elective surgery was defined as procedures expected to last more than two hours or with an anticipated blood loss greater than 500 millilitres. Patients in whom the planned surgery included any of the following surgical procedures were eligible for recruitment:

- Orthopaedic surgery: revision hip arthroplasty, total hip replacement, total knee replacement, fusion/instrumentation of multiple lumbar or thoracic vertebrae);
- General abdominal surgery: laparotomy including partial hepatectomy, pancreatic surgery, re‐operative colon surgery, abdomino-perineal resections, anterior resections, pan-proctocolectomies, hepatobiliary bypass procedures);
- Urological surgery (radical prostatectomy, radical cystectomy, radical nephrectomy).

### Cohort 2

In the second study, the definition of major elective surgery was broadened to include patients undergoing the following vascular surgical procedures: aortic aneurysm repair, carotid endarterectomy, arterial reconstruction, amputation for vascular disease. Data were collected on patients who fulfilled these extended criteria and who underwent surgery between 2 March 2004 and 29 March 2005 (when the Surgical Outcomes Research Centre suspended work).
