# Supplementary material for: Survival after postoperative morbidity: a longitudinal observational cohort study
Source: Br J Anaesth. 2014 Jul 10;113(6):977–84. doi: 10.1093/bja/aeu224 (PMC4235571; doi:10.1093/bja/aeu224)
Supplement: Supplementary Data [file supp_aeu224_aeu224supp_app2.docx]

| Variable | Hazard Ratio | Standard error | p value | 95% C.I. |
| --- | --- | --- | --- | --- |
| Patient /Surgical Risk factors | | | | |
| P-POSSUM (%) | 1.05 | 0.01 | <0.001 | 1.03 - 1.07 |
| Cancer | 2.65 | 0.39 | <0.001 | 1.99-3.53 |
| Age (years) | 1.03 | 0.00 | <0.001 | 1.02 - 1.04 |
| Male Gender | 1.05 | 0.12 | 0.65 | 0.85 - 1.30 |
| General surgery | 2.12 | 0.63 | 0.01 | 1.19 – 3.79 |
| Vascular surgery | 1.77 | 0.47 | 0.03 | 1.05 – 2.98 |
| POMS defined morbidity domains | | | | |
| Pulmonary | 0.94 | 0.14 | 0.67 | 0.71 - 1.25 |
| Infection | 1.07 | 0.59 | 0.58 | 0.84 - 1.37 |
| Renal | 0.94 | 0.12 | 0.64 | 0.73 - 1.20 |
| Gastrointestinal | 0.78 | 0.12 | 0.11 | 0.57 - 1.06 |
| Cardiac | 1.13 | 0.21 | 0.52 | 0.71 - 1.63 |
| Neurological | 1.64 | 0.37 | 0.03 | 1.06 – 2.54 |
| Haematological | 1.09 | 0.19 | 0.61 | 0.78 - 1.52 |
| Pain | 1.12 | 0.17 | 0.45 | 0.83 - 1.50 |
| Final Morbidity Day 3 | 1.58 | 0.32 | 0.02 | 1.06 - 2.36 |
| Final Morbidity Day 5 | 1.64 | 0.36 | 0.03 | 1.07 - 2.52 |
| Final Morbidity Day 8 | 1.87 | 0.32 | <0.01 | 1.20 – 2.94 |
| Final Morbidity day 15 + post-op Year 1 | 5.77 | 1.82 | <0.001 | 3.11 – 10.73 |
| Final Morbidity day 15 + post-op year 2-3 | 4.01 | 1.21 | <0.001 | 2.22 - 7.73 |
| Final Morbidity Day 15 + post-op year ≥ 4 | 2.22 | 0.64 | <0.01 | 1.26 – 3.92 |
| Interactions between types of surgery and POMS defined morbidity | | | | |
| General / GI morbidity | 0.68 | 0.22 | 0.23 | 0.36 -1.28 |
| Vascular / Cardiac Morbidity | 1.91 | 0.82 | 0.13 | 0.83 – 4.41 |

**Appendix 2: Unrestricted model**
